# A seven-year longitudinal study of the association between neurocognitive function and basic self-disorders in schizophrenia

**DOI:** 10.3389/fpsyg.2023.1124859

**Published:** 2023-02-27

**Authors:** Elisabeth Haug, Merete G. Øie, Ingrid H. Svendsen, Paul Møller, Barnaby Nelson, Ingrid Melle

**Affiliations:** ^1^Department of Acute Psychiatry and Psychosis Treatment, Innlandet Hospital Trust, Brumunddal, Norway; ^2^Department of Research, Innlandet Hospital Trust, Brumunddal, Norway; ^3^Department of Psychology, University of Oslo, Oslo, Norway; ^4^Clinic for Mental Health and Addiction, Vestre Viken Hospital Trust, Parkville, VIC, Australia; ^5^Centre for Youth Mental Health, The University of Melbourne, Parkville, VIC, Australia; ^6^Orygen, Parkville, VIC, Australia; ^7^Norment Centre, University of Oslo, Oslo, Norway; ^8^Norment Centre, Oslo University Hospital, Oslo, Norway

**Keywords:** executive function, psychoses, phenomenology, verbal memory, self disturbance

## Abstract

**Introduction:**

Basic self-disorders (SDs) and neurocognitive impairments are fundamental trait-like aspects of schizophrenia spectrum disorders. There has been little research on the association between SDs and neurocognitive impairments in schizophrenia, and no longitudinal studies have investigated if they are related. The aim of this study was to investigate the association between SDs and neurocognitive function in a follow-up study of patients with schizophrenia.

**Methods:**

SDs and neurocognition were examined in 35 patients with schizophrenia during their first treatment and 7 years later (mean 7.1, SD 0.42). SDs were examined with the Examination of Anomalous Self-Experience (EASE) instrument. The neurocognitive examination included assessments of psychomotor speed, executive- and memory functions.

**Results:**

Poorer executive functions at baseline were significantly associated with more SDs 7 years later and smaller reductions in SDs over time. There were no significant associations between other neurocognitive functions and SDs.

**Discussion:**

Executive functions are important for self-regulation, and impairments in these functions in everyday life may have an impact on the development and/or persistence of SDs.

## 1. Introduction

Although both basic self-disorders (SDs) and neurocognitive deficits are core aspects of schizophrenia, there has been little research on the association between them, and results so far have been inconsistent (Haug et al., 2012; Nordgaard et al., 2015; Koren et al., 2019; Nelson et al., 2020; Hernandez-Garcia et al., 2021; Trask et al., 2021). An integration of findings across the phenomenological domains, including SDs, and neurocognitive domains, could represent an advance in the understanding of schizophrenia and could possibly be beneficial for intervention strategies (Nelson et al., 2019, 2020).

Prognosis, everyday functioning, and long-term outcome may all be affected by neurocognitive impairments (Keefe and Harvey, 2012; Lepage et al., 2014). Persons with first-episode schizophrenia spectrum disorders have been found to exhibit impairments in executive functions, verbal learning and memory, psychomotor speed, and attention (Townsend and Norman, 2004; Skelley et al., 2008; McCutcheon et al., 2023). Most research indicates that neurocognitive deficits stay stable (Palmer et al., 2009; De Herdt et al., 2013; Rund et al., 2016). However, some studies have reported a decline in neurocognitive functions after the first-episode psychosis (Fett et al., 2019; Zanelli et al., 2019). Neurocognitive impairments in schizophrenia are features more associated with the broadly- and persistently observed impairments in everyday functioning than psychotic symptoms (Lepage et al., 2014).

SDs are disturbances of the person’s feeling of identity and vitality and the experience of belonging in the real world (Parnas and Handest, 2003; Parnas et al., 2005). They are disturbances at the basic levels of consciousness. Depersonalization, self-alienation, difficulties in grasping familiar meanings, hyperreflexivity, social interaction difficulties, unusual bodily sensations, and existential reorientation are all aspects of SDs (Parnas and Handest, 2003; Sass and Parnas, 2003). Studies have demonstrated significantly more SDs in schizophrenia spectrum disorders than in other psychiatric disorders. SDs are found to have a trait-like quality and are thus considered core features/characteristics of these disorders. SDs are not considered a result of psychosis but instead seem to generate psychopathology in schizophrenia spectrum disorders (Nordgaard and Parnas, 2014).

In an earlier report from the baseline assessment of the current cohort of patients with schizophrenia, our research group found few associations between SDs and neurocognitive functions in general. The only significant association was between high levels of SDs and impaired immediate verbal memory (Haug et al., 2012). A pilot study among non-psychotic adolescents who were help-seeking for mental problems also found a weak association between SDs and the summary scores of the neurocognitive domains of executive functions, verbal memory, theory of mind, and emotion recognition (Koren et al., 2019). However, a study of patients at risk of developing schizophrenia did not find any associations between neurocognitive function and SDs (Comparelli et al., 2016). Two recent studies have explored the associations between SDs and neurocognitive function in schizophrenia, using a self-assessment instrument with five subscales (IPASE) to measure SDs. One of the studies revealed a significant relationship between certain domains of general cognition deficits and SDs (Hernandez-Garcia et al., 2021), and the other study found that the IPASE-Cognition subscale was negatively associated with neurocognitive functioning (Trask et al., 2021). Two studies by another research group did not find any associations between neurocognitive functions and SDs in patients with schizophrenia (Nordgaard et al., 2015; Sandsten et al., 2022).

Studies using more experimental cognitive tests that are designed to measure unique aspects of memory and perception have also found some associations between these functions and SDs.

Nelson et al. did find a significant association between SDs and source monitoring deficits (Nelson et al., 2019, 2020). Source monitoring deficits refer to difficulties in making attributions about the origins of mental experiences. This is by definition closely overlapping with certain core SDs features, such as for instance; that the persons own thoughts, feelings or actions appear as if they were not generated by him or herself (disturbed first-person perspective).

The psychopathology of the bodily self (i.e., disturbances in recognition the “self-body as distinct from body of others”) and abnormal space experiences are also phenomena closely linked to and partly overlapping SDs (Gallese and Ferri, 2014; Stanghellini et al., 2020). The loss of the implicit functioning of the body in everyday life may lead to inability to interrelate with others (Fuchs, 2005; Stanghellini et al., 2011). A study showed that implicit knowledge about the bodily self was impaired and that self-other discrimination was problematic in first-episode schizophrenia patients (Ferri et al., 2012). Further, abnormal space experiences have also been found common in persons with schizophrenia and refers to the experiences of altered perception and distorted sense of space (Stanghellini et al., 2020). A study using the Enfacement Illusion test (Tsakiris, 2008) to examine the effect of multisensory integration on self-recognition found that a group of patients with schizophrenia differed from healthy controls in their self-recognition. This suggests that temporal factors may affect how multisensory stimuli are integrated with self-related stimuli in schizophrenia (Sandsten et al., 2020).

We do not know any longitudinal studies investigating the possible associations between SDs and neurocognitive functions in schizophrenia.

Neurocognitive functions sub-serve consciousness, and the basic self is an integral feature of conscious experience, including the sense of unity in the conscious experience that arises from integrating affect, will, and volition (Fabrega, 1989). Therefore, it would be surprising if a disturbance of the basic self does not have some neurocognitive correlates.

The current study aimed to explore the relationship between:

1. Neurocognitive performance at first treatment and SDs at 7 years follow-up.
2. Neurocognitive performance and SDs at 7 years follow-up.
3. Neurocognitive performance at first treatment and change in SDs over 7 years.

Our hypotheses were that:

1. Poorer neurocognitive functions *at baseline* would be associated with high levels of SDs at follow-up.
2. Poorer neurocognitive functions *at follow-up* would be associated with high levels of SDs at follow-up.
3. Poorer neurocognitive functions *at baseline* would be associated with less reduction in SDs from baseline to follow-up.

Deficiencies in the patients’ ability to grasp, direct, remember, and reason about their thoughts may be associated with verbal memory deficits. These functions are related to SDs, and impaired immediate verbal memory was significantly associated with SDs in this sample at baseline (Haug et al., 2012). Further, executive functions are essential for regulating thought, emotion, and behavior. Thus, we hypothesized that impaired executive functions and impaired immediate verbal memory at baseline would be associated with less reduction in SDs from baseline to follow-up and more SDs at follow-up. Further, we expected that impaired executive functions and immediate verbal memory at follow-up would also be associated with more SDs at that point in time.

## 2. Materials and methods

### 2.1. Design and sample

**Figure 1:**
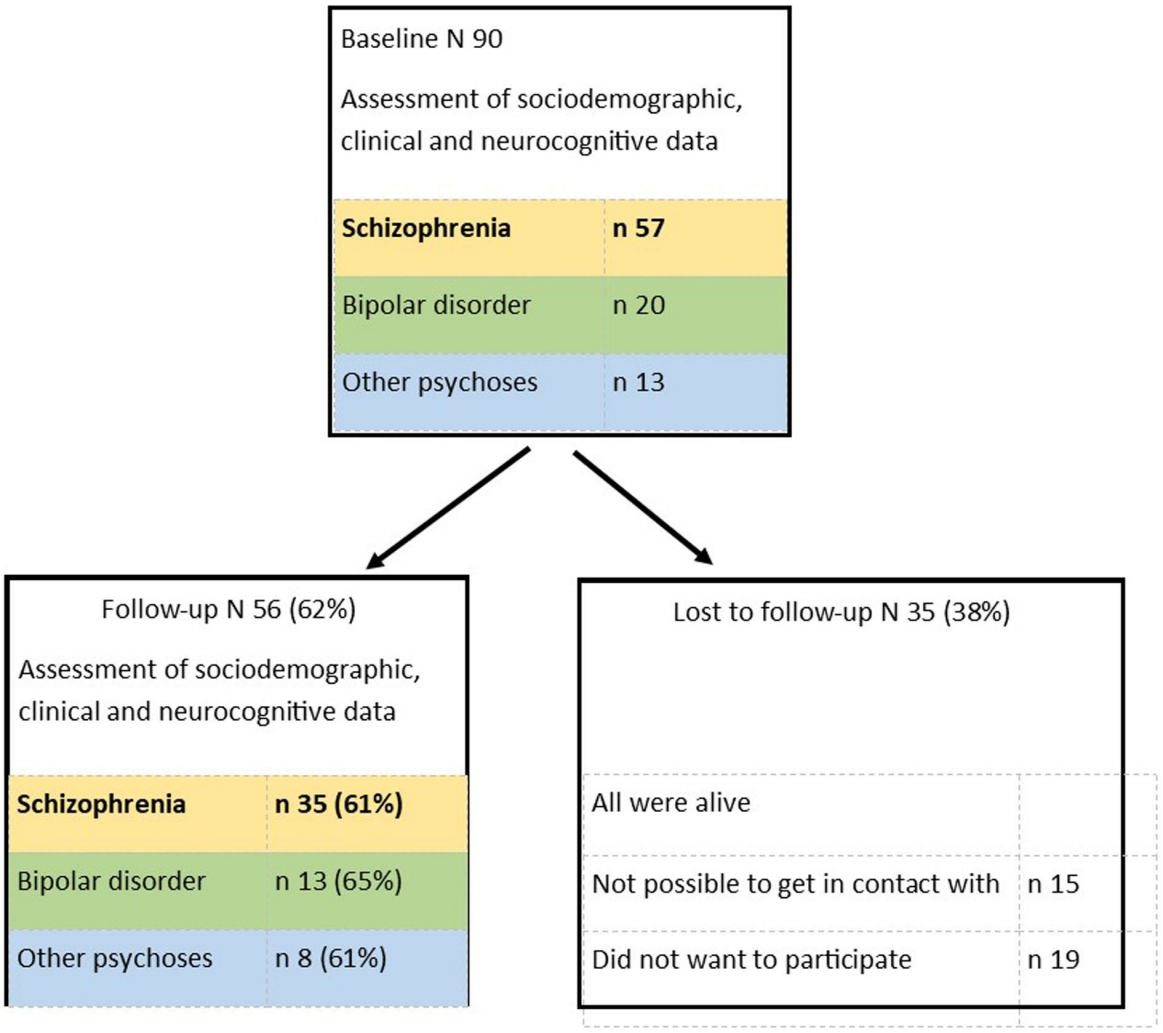
Study flow.

This study is a seven-year follow-up study of 57 patients with first-episode schizophrenia in two neighboring Norwegian counties. For a description of the baseline study, including inclusion and exclusion criteria, see Haug et al. (2012). In the follow-up study, 35 patients (61%) agreed to participate. For a description of the follow-up study, see Svendsen et al. (2018). All participants provided informed consent to participate at both time points. The study was approved by the Regional Committee for Medical Research Ethics and the Norwegian Data Inspectorate (Figure 1).

### 2.2. Clinical assessments at baseline and follow-up

Diagnoses were set by using the Structured Clinical Interview for DSM-IV Axis I disorders (SCID module I, chapter A–E; First et al., 1996), with additional information from medical charts.

The interview for the Positive and Negative Syndrome Scale (SCI-PANSS) (Kay et al., 1987) was used to measure psychotic symptoms. The use of antipsychotics at baseline and follow-up was measured as Defined Daily Dosages (DDD; WHO, 2023).

### 2.3. Neurocognitive assessment

Clinical psychologists conducted the baseline assessments, and two trained research assistants performed the follow-up assessments. An experienced neuropsychologist and researcher (MØ) trained and supervised them. All subjects were examined separately, and the neurocognitive tests were given in the same order. The patients were tested when they were clinically stabilized and not in an acute episode of illness. At baseline, a more comprehensive test battery was used. The total time for all assessments was approximately 3 h at baseline and 2 h at follow-up. The current estimated IQ at baseline was assessed with the Wechsler Abbreviated Scale of Intelligence (Wechsler, 2007). The remaining tests cover domains that are sensitive to neurocognitive dysfunction in psychosis (Green et al., 2004):

**Psychomotor speed**: Digit Symbol from Wechsler Adult Intelligence Scale (WAIS-III; Wechsler, 1997; Wechsler, 2003). For 120 s, the participant has to fill in blank spaces with the symbol corresponding to the number above the blank space as rapidly as possible. The number of squares correctly filled in determines the score.

**Immediate verbal memory**: Part 1 of the Logical Memory Test from the Wechsler Memory Scale [WMS] III (Wechsler et al., 2008). In this test, the subjects are required to immediately recall details of two short verbal stories.

**Executive functions**: Letter Number Span from WAIS-III (Wechsler, 1997, 2003). The task involves hearing a series of letters and digits and then reporting back the stimuli in alphabetical order and ascending numerical order. The result is a total of correctly recalled trials. This task requires *auditory working memory*.

We included selected measures from the Delis-Kaplan Executive Function System (D-KEFS; Delis et al., 2001). In the Colour-Word Interference Test (CWIT), Condition 3, the participant has to inhibit an overlearned verbal response when naming dissonant ink colors in which the words are printed. In CWIT Condition 4, the person has to switch back and forth between naming the dissonant ink colors and reading the words. Condition 3 measures *inhibition*, and condition 4 measures *cognitive flexibility*. Completion time in seconds was examined.

For all tests, standard scores or T-scores according to norms were used (See Table 1).

**Table 1:** Demographic and clinical characteristics.

	Baseline	Follow-up	Paired Samples Test (*p*)
Number of patients	35	35	
Demographics			
Male gender, *n* (%)	17 (49)		
Age years, mean (SD)	25.5 (7.8)	32.5 (7.7)	
Education years, mean (SD)	12.3 (2.2)		
DUP weeks, median (range)	104 (4–1,040)		
Medication use			
Using antipsychotics (*N* %)	25 (71%)	17 (49%)	
DDD for those using (median/range)	0.62 (1.38)	1.0 (2.70)	
Symptoms, mean (SD)			
PANSS total score, mean (SD)	75.4 (17.6)	37.9 (9.9)	<0.001
Self disorders (SDs)			
EASE total, mean (SD)	23.2 (9.6)	14.7 (9.1)	<0.001
EASE domain 1, mean (SD)	8.7 (3.2)	6.5 (3.5)	0.01
EASE domain 2, mean (SD)	7.5 (3.6)	4.5 (3.5)	<0.001
EASE domain 3, mean (SD)	2.8 (2.1)	1.7 (1.8)	0.001
EASE domain 4, mean (SD)	1.4 (1.2)	0.8 (1.1)	0.016
EASE domain 5, mean (SD)	2.9 (2.1)	1.2 (1.8)	<0.001
Functional level, mean (SD)			
GAF symptom	33.7 (7.1)	51.2 (15.6)	<0.001
GAF function	35.5 (4.7)	55.1 (15.9)	<0.001
Neurocognition*			
Psychomotor speed^a^ (S-score) mean (SD)	6.9 (2.4)	8.8 (9.0)	0.351
Verbal immediate memory^b^ (S-score) mean (SD)	8.1 (2.8)	9.2 (3.4)	<0.001
Executive functions (S-score)	7.6 (2.5)	8.1 (2.2)	0.010
Working memory^c^ mean (SD)	8.0 (3.2)	7.3 (5.0)	0.001
	7.1 (3.7)	5.2 (4.9)	0.017
Inhibition^d^ mean (SD)			
Cognitive flexibility^d^ mean (SD)			
Estimated IQ, mean (SD)	92.5 (14.7)		
WASI Verbal IQ	98.4 (12.7)		
WASI Performance IQ			
WASI FIQ4 subtests^e^	95.0 (14.0)		

SDs at follow-up refer to the last 2 years.

* For the normal population mean S-score = 10 (SD = 3).

a Digit Symbol from WAIS-III.

b Logical Memory Test Wechsler Memory Scale [WMS] III.

c Letter Number Span from WAIS-III.

d The Color-Word Interference subtests from the Delis-Kaplan Executive Function System (D-KEFS).

e Total IQ.

### 2.4. Assessment of SDs

SDs were assessed with the Examination of Anomalous Self-Experience (EASE) (Parnas et al., 2005), a standard measure focusing specifically on SDs, comprising five domains: 1. Cognition and stream of consciousness. 2. Self-awareness and presence. 3. Bodily experiences. 4. Demarcation/transitivism. 5. Existential reorientation. For a detailed description of this assessment, see Svendsen et al. (2018).

The EASE manual usually aims to capture the lifetime experiences of SDs, and at baseline, we registered only lifetime experiences. *Since we aimed to measure change and stability in SDs at follow-up, we rated SD experiences during the last 2 years separately from lifetime experiences at follow-up. We used the information about the last 2 years in the current analyses.* EASE change was calculated as EASE at baseline minus EASE at follow-up.

### 2.5. Statistical analyses

All analyses were performed with the statistical package SPSS, version 28.0 (SPSS Inc., Chicago, IL, United States). Mean and standard deviations are reported for continuous variables and percentages for categorical variables. All variables of interest were examined for deviations from normality. Neurocognitive indices and EASE scores at both time points were normally distributed. Antipsychotic use at both time points were skewed, and were transformed to their natural logarithm before they were entered into parametric analyses. Partial correlation analyses were used to investigate possible associations between neurocognitive measures at baseline and EASE change and EASE total score at follow-up, corrected for the use of antipsychotics. Paired sample statistics were used to measure differences in neurocognitive function and SDs from baseline to follow-up.

## 3. Results

Sociodemographic and clinical data are shown in Table 1.

There was a significant decrease in the level of SDs from baseline to follow-up, with EASE total scores and all domain scores decreasing (Table 1). There was a significant increase in immediate verbal memory and working memory and a significant decrease in inhibition and cognitive flexibility from baseline to follow-up.

Poorer executive functions (inhibition and cognitive flexibility) at baseline were significantly associated with high levels of SDs at follow-up. Partial correlations corrected for antipsychotic use at baseline were −0.411*, *p* = 0.016 for inhibition, and −0.498**, *p* = 0.003 for cognitive flexibility, and partial correlations corrected for antipsychotic use at follow-up were − 363*, *p* = 0.035 for inhibition, and −0.466**, *p* = 0.006 for cognitive flexibility. This confirms the hypothesis that poorer neurocognitive functions *at baseline* would be associated with high levels of SDs at follow-up.

In comparison, better executive functions (working memory and cognitive flexibility) at baseline were significantly associated with decreased EASE total scores from baseline to follow-up. Partial correlations corrected for antipsychotic use at base line were 0.514**, *p* = 0.002 for working memory, and 0.474**, *p* = 0.005 for cognitive flexibility. Partial correlations corrected for antipsychotic use at follow-up were.487**, *p* = 0.005 for working memory, and.472**, *p* = 0.005 for cognitive flexibility. This confirms the hypothesis that poorer neurocognitive functions *at baseline* would be associated with less reduction in SDs from baseline to follow-up.

There were no associations between neurocognitive function at follow-up and SDs at follow-up, which disproves the hypothesis that poorer neurocognitive functions *at follow-up* would be associated with high levels of SDs at follow-up.

**Figure 2:**
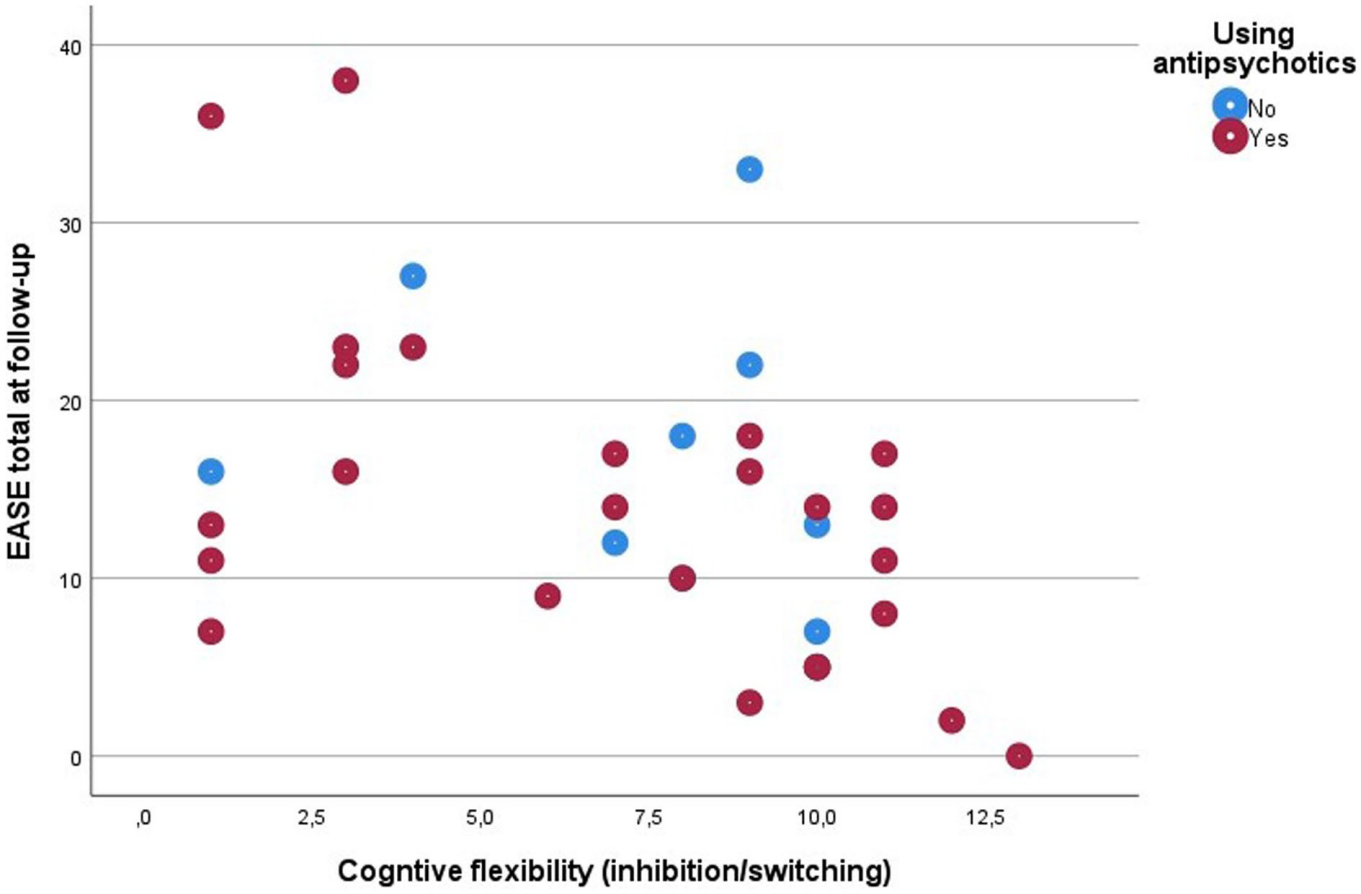
Scatter plot of associations between cognitive flexibility and SDs.

**Figure 3:**
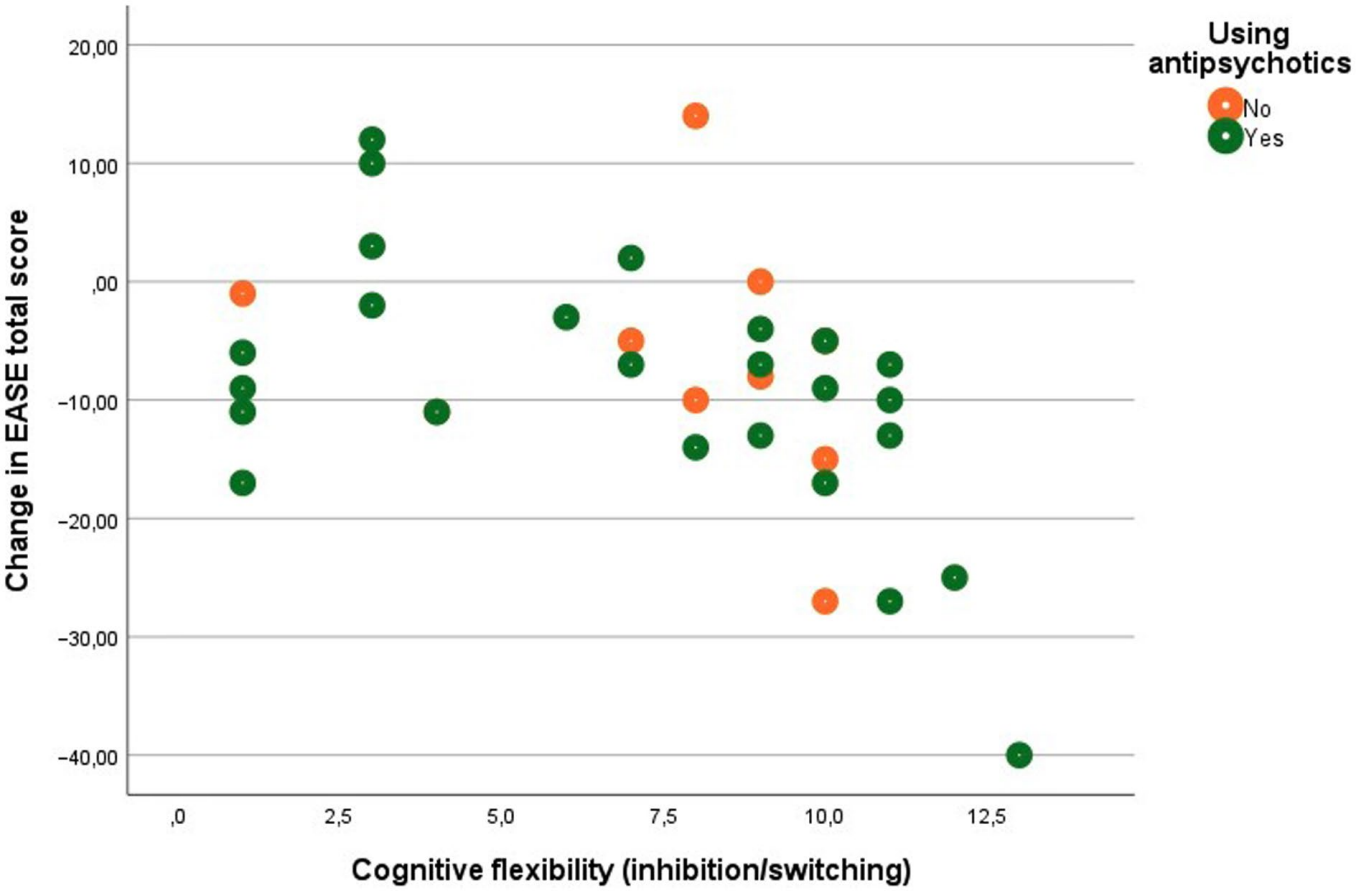
Scatter plot of associations between cognitive flexibility and SDs.

In the baseline study of the current cohort (*N* = 57) we did find an association between SDs and verbal memory (Haug et al., 2012), but in the baseline assessment of the present sub-sample that we were able to reassess at follow-up (*N* = 35) we did not find any associations between neurocognitive function and SDs (Figures 2, 3; Table 2).

**Table 2:** Partial correlations between neurocognition at baseline and SDs at follow-up and change in SDs.

Correcting for antipsychotic use (DDD) at baseline		
Neurocognitive functions (baseline)	EASE total score (follow-up)	EASE change
Psychomotor speed^a^	−0.325 *p* = 0.061	0.291 *p* = 0.094
Verbal memory^b^	−0.314 *p* = 0.071	0.014 *p* = 0.938
Executive functions		
Working memory^c^	−0.347* *p* = 0.044	0.514** *p* = 0.002
Inhibition^d^	−0.411* *p* = 0.016	0.318 *p* = 0.067
Cognitive flexibility^d^	−0.498** *p* = 0.003	0.474** *p* = 0.005
Correcting for antipsychotic use (DDD) at follow-up.		
Neurocognitive functions (baseline)	EASE total score (follow-up)	EASE change
Executive functions		
Working memory^c^	−0.333 *p* = 0.054	0.487** p = 0.005
Inhibition^d^	−0.363* *p* = 0.035	0.292 p = 0.094
Cognitive flexibility^d^	−0.466** *p* = 0.006	0.472** p = 0.005

* Correlation is significant at the .05 level (2-tailed).

** Correlation is significant at the .01 level (2-tailed).

a Digit Symbol from WAIS-III.

b Logical Memory Test Wechsler Memory Scale [WMS] III.

c Letter Number Span from WAIS-III.

d The Color-Word Interference subtests from the Delis-Kaplan Executive Function System (D-KEFS).

## 4. Discussion

### 4.1. General discussion

Our main findings were that poorer baseline executive functions (inhibition and cognitive flexibility) in patients with schizophrenia were associated with higher levels of SDs at 7 years of follow-up. Further, at baseline, better executive functions, particularly cognitive flexibility and working memory were associated with a more significant decrease in SDs during the follow-up period. We also found that immediate verbal memory and psychomotor speed were not associated with changes in SDs or SDs at follow-up. In the previous cross-sectional baseline study with more patients included (*N* = 57), however, we found that high levels of SDs were significantly associated with poorer verbal immediate memory (Haug et al., 2012). We hypothesized that deficiencies in immediate verbal memory could lead to problems understanding, directing, remembering, and reasoning about one’s thoughts and self-knowledge, functions linked to various aspects of SDs. However, our present follow-up results indicate that immediate memory difficulties are less critical for how SDs develop over time. Instead, we found that executive dysfunctions at baseline were associated with higher and more stable SDs over time (i.e., less reduction in SDs over time and higher SDs scores at follow-up). Short-term (immediate) memory and working memory have been used to refer to the maintenance and the maintenance plus manipulation of information, respectively. However, correlational studies have been unable to distinguish the two constructs consistently, and there is evidence of a significant, if not complete, overlap (Aben et al., 2012). *Thus, working memory is* not entirely distinct from short-term or immediate memory as it includes short-term memory (Cowan, 2008).

We expected that SDs should have some form of neurocognitive correlates, but we found very few significant correlations. However, phenomenological psychopathology is a purely subjective, experiential scientific domain of mental life, arising exclusively from patients’ self-reports, and only possible to assess through in-depth interviews. The neurocognitive fields of psychopathology are, on the contrary, test-based, objectively assessed sets of mental functions. This fact may leave its imprint on potential relationships between the two scientific domains.

Executive functions generally allow us to anticipate outcomes and adapt to changing life situations and are essentially the conscious regulation of thought, emotion, and behavior (Diamond, 2013). These functions help us focus our attention, resist distraction, preserve flexible thinking, set-shifting, and keep a thought or an image in mind (Prebble et al., 2013; Alderson-Day and Fernyhough, 2015). They provide the capacity to bind experiences across time, to perceive the present moment as both a continuation of our past and as a prelude to our future (Barkley, 2004; Prebble et al., 2013). Thus, executive functions sub-serve aspects of self-regulation, and impairments in executive functions may be associated with impairing aspects of the self’s ability to function and maintain a sense of constancy and continuity. It is reasonable to believe that the executive sub-functions impact “continuity” in the sense of selfhood, emotional self-regulation, and inner speech (Prebble et al., 2013; Alderson-Day and Fernyhough, 2015).

The executive function tasks used in the interference-test in this study require a weighting in favor of “top down” control. Hierarchical predictive processing has recently gained substantial attention as a computational neuroscientific approach to psychotic symptoms (Fletcher and Frith, 2009; Seth, 2013; Powers et al., 2017) and has also been applied to SDs (Seth et al., 2011; Clowes and Gärtner, 2018; Sass et al., 2018). On this account, top-down signals convey predictions based on prior expectations, while bottom-up signals convey prediction errors. The brain uses previously generated high-level causal models to attempt to predict sensory activity continually. The prediction error is the discrepancy between the reward and its prediction. The precision-weighting of predictions and prediction error is mediated by noradrenergic, cholinergic, glutamatergic, and gamma-aminobutyric acidergic function (Powers et al., 2016). Difficulties with the balance and weighting of top-down and bottom-up processing may have a role in the onset and maintenance of psychotic symptoms and SDs, according to new models (Clowes, 2018; Clowes and Gärtner, 2018; Nelson et al., 2019). The current findings, which are based on the relationship between EASE scores at follow-up and the interference test, provides the first direct empirical data that predictive coding (specifically, disturbance in the “top-down,” prior-based interpretation of information) may play a role in SDs, a finding worthy of further investigation.

Executive dysfunctions were not related to SDs at baseline in our patients. However, poorer executive functions at baseline were associated with more SDs at follow-up. Because these functions are essential for self-regulation, extended and persistent impairments in executive functions in everyday life may reflect and influence the development of SDs over time. Although we found associations between executive functions at baseline and SDs at follow-up, there may be mediating or moderating factors that we did not investigate. We do not know any previous studies investigating the longitudinal relationship between neurocognitive impairments and SDs, and further studies are necessary to generate more knowledge regarding this topic. The current study sample is small, with the possibility of finding clearer associations between neurocognitive impairments and SDs in the context of larger samples. Future work should also look at a broader range of neuropsychological functions such as aberrant salience, time awareness, source monitoring, hierarchical predictive processing, and multisensory integration – i.e., go beyond the “standard” neuropsychological battery (Nelson et al., 2019). Future findings across the phenomenological and neurocognitive domains could represent a significant advance in understanding schizophrenia and potentially improve early identification and intervention strategies.

### 4.2. Strengths and limitations of the study

#### 4.2.1. Strengths

(1) At follow-up assessment of SDs and neurocognitive functions, the testers were blind to the baseline data; (2) we enrolled patients consecutively from all treatment facilities in a large combined rural/urban catchment area, so the study population is highly representative of patients in this catchment area.

#### 4.2.2. Limitations

The sample size of patients at follow up was modest, with some attrition from the sample recruited at baseline. However, the baseline demographic and clinical features of individuals who participated and those who did not participate in the follow-up were not significantly different. There is no indication of a systematic bias in participants available for follow-up. The patients had also received variable types of treatment over the follow-up period, which may have affected the results. It has been documented that antipsychotic medication has a minimal effect on neurocognition (Keefe and Harvey, 2012).

### 4.3. Conclusion

In conclusion, executive functions may influence the development of SDs, the core phenomenological features of schizophrenia, over time, and possibly the other way around.

## Data availability statement

The raw data supporting the conclusions of this article will be made available by the authors, without undue reservation.

## Ethics statement

The studies involving human participants were reviewed and approved by REK, Regionale komiteer for medisinsk og helsefaglig forskningsetikk. The patients/participants provided their written informed consent to participate in this study.

## Author contributions

EH, MØ, PM, and IM planned the original study, while EH, MØ, IM, BN, and IS planned the follow-up study. IS and EH conducted the follow-up. IS conducted the follow-up interviews. EH did the statistical analyses and wrote the first draft of the paper. All authors contributed to the article and approved the submitted version.

## Funding

This work was supported by Innlandet Hospital Trust, Norway (Grant nos. 150281 and 150338), and Eastern Norway Health Authority (Grants no. 2006258-2011085-2014102) supporting EH and IS. BN was supported by an NHMRC Senior Research Fellowship (Grant no. 1137687).

## Conflict of interest

The authors declare that the research was conducted in the absence of any commercial or financial relationships that could be construed as a potential conflict of interest.

## Publisher’s note

All claims expressed in this article are solely those of the authors and do not necessarily represent those of their affiliated organizations, or those of the publisher, the editors and the reviewers. Any product that may be evaluated in this article, or claim that may be made by its manufacturer, is not guaranteed or endorsed by the publisher.
